# H3N2 influenza hemagglutination inhibition method qualification with data driven statistical methods for human clinical trials

**DOI:** 10.3389/fimmu.2023.1155880

**Published:** 2023-04-06

**Authors:** Sheetal Sawant, Sarah Anne Gurley, R. Glenn Overman, Angelina Sharak, Sarah V. Mudrak, Thomas Oguin, Gregory D. Sempowski, Marcella Sarzotti-Kelsoe, Emmanuel B. Walter, Hang Xie, Marcela F. Pasetti, M. Anthony Moody, Georgia D. Tomaras

**Affiliations:** ^1^ Center for Human Systems Immunology, Department of Surgery, Duke University, Durham, NC, United States; ^2^ Duke Human Vaccine Institute, Duke University, Durham, NC, United States; ^3^ Department of Immunology, Duke University, Durham, NC, United States; ^4^ Department of Pediatrics, Duke University, Durham, NC, United States; ^5^ Duke Global Health Institute, Duke University, Durham, NC, United States; ^6^ Division of Viral Products, Office of Vaccines Research and Review, Center for Biologics Evaluation and Research, U.S. Food and Drug Administration, Silver Spring, MD, United States; ^7^ Department of Pediatrics, University of Maryland School of Medicine, Baltimore, MD, United States; ^8^ Center for Vaccine Development, University of Maryland School of Medicine, Baltimore, MD, United States

**Keywords:** hemagglutination inhibition (HAI), influenza, qualification, antibody, statistical analysis, data pipeline, human, clinical trials

## Abstract

**Introduction:**

Hemagglutination inhibition (HAI) antibody titers to seasonal influenza strains are important surrogates for vaccine-elicited protection. However, HAI assays can be variable across labs, with low sensitivity across diverse viruses due to lack of standardization. Performing qualification of these assays on a strain specific level enables the precise and accurate quantification of HAI titers. Influenza A (H3N2) continues to be a predominant circulating subtype in most countries in Europe and North America since 1968 and is thus a focus of influenza vaccine research.

**Methods:**

As a part of the National Institutes of Health (NIH)-funded Collaborative Influenza Vaccine Innovation Centers (CIVICs) program, we report on the identification of a robust assay design, rigorous statistical analysis, and complete qualification of an HAI assay using A/Texas/71/2017 as a representative H3N2 strain and guinea pig red blood cells and neuraminidase (NA) inhibitor oseltamivir to prevent NA-mediated agglutination.

**Results:**

This qualified HAI assay is precise (calculated by the geometric coefficient of variation (GCV)) for intermediate precision and intra-operator variability, accurate calculated by relative error, perfectly linear (slope of -1, R-Square 1), robust (<25% GCV) and depicts high specificity and sensitivity. This HAI method was successfully qualified for another H3N2 influenza strain A/Singapore/INFIMH-16-0019/2016, meeting all pre-specified acceptance criteria.

**Discussion:**

These results demonstrate that HAI qualification and data generation for new influenza strains can be achieved efficiently with minimal extra testing and development. We report on a qualified and adaptable influenza serology method and analysis strategy to measure quantifiable HAI titers to define correlates of vaccine mediated protection in human clinical trials.

## Introduction

Influenza A (H3N2) has become a predominant circulating subtype post the 2009 H1N1 pandemic and is therefore a focus of influenza vaccine research (1). Seasonal influenza vaccines led to decreases in infection but the elderly, immunocompromised individuals, and individuals with chronic illnesses remain at risk for severe infection and young children and adults without pre-existing immunity could also be vulnerable to novel influenza strains (e.g., the 2009 H1N1 pandemic) (2, 3). The emergence of highly pathogenic avian influenza and other zoonotic influenza viruses poses a continued threat to the public health. Therefore, improved prevention and management approaches for seasonal and pandemic influenza across all populations are urgently needed, including the development of new universal vaccines that offer broad and durable protection (4).

The National Institute of Allergy and Infectious Diseases (NIAID) established the Collaborative Influenza Vaccine Innovation Centers (CIVICs) program. The CIVICs program is a network of research centers and cores that work together to advance the production and clinical testing of improved seasonal and universal influenza vaccines. The most promising vaccine candidates are advanced into Phase I and II clinical trials. The Duke Center for Human Systems Immunology (CHSI) as part of the CIVICs program standardizes and qualifies endpoint serology assays such as the hemagglutination inhibition (HAI) assay for evaluating influenza specific antibodies in CIVICs clinical trials and controlled human challenge studies.

The HAI assay is the most utilized canonical method to quantify influenza specific antibodies for influenza vaccination clinical studies. The principle of the HAI method is to take advantage of the ability of the influenza HA proteins to bind to, or agglutinate, the sialic acids on avian or mammalian RBCs. The agglutination by the HA proteins holds RBCs into a lattice formation and prevents their precipitation. The HA-guinea pig RBC lattice appears as a cloudy pink haze in the microtiter plate well as opposed to the halo morphology typical of precipitated un-agglutinated guinea pig RBCs. HA-specific antibodies can block the formation of HA/RBC lattice resulting in the precipitation of un-agglutinated guinea pig RBCs. The antibody titer corresponds to the inverse of the serum dilution of the last well that contains an RBC precipitate similar in size and morphology to the RBC control wells. The accurate and precise measure of antibody titers through the HAI assay contributes to advancing vaccine research by enabling a quantitative comparison of antibodies elicited by different vaccine regimens. For example, HAI antibody titers to strains predicted to be predominant in the next flu season can be used to estimate vaccine efficacy in simulated vaccine trials (5). Moreover, HAI antibody titers have been used in clinical investigations to evaluate vaccine immunogenicity and predict the proportion of vaccine induced protection (3, 6). It is accepted by regulatory agencies for vaccine licensure.

As the HAI assay must be standardized for each specific influenza virus strain, controlling for technical variables such as type and concentration of red blood cells, incubation times, positive and negative controls and the specialized expertise required to determine HAI antibody titers by visual observation, comparison of results across laboratories can be difficult. Method qualification or validation, which includes development of standardized protocols and establishes the parameters of the assay, can be used to ensure reliable and reproduceable results across laboratories (7, 8). Multiple groups (7, 9–11) have reported on the standardization, qualification, validation and optimization of an HAI assay, and we provide here a concise tabulated summary comparing assay designs and analysis methods made available by these research groups, as well as those presented by our team (**Table 1**).

**Table 1:** Table comparing the previous literature on HAI assay development efforts in terms of assay design, data processing and statistical analysis details.

Details about	2009 HAI Qualification (7)	2011 WHO Manual (12)	2013 Validation of Modified HAI (10)	2016 HAI Standardization (11)	2017 HAI Optimization (9)	2023 CIVICs HAI Qualification
Serum starting dilution	1:10	1:10	1:8	1:10	1:8	1:10
RDE-treatment and heat inactivation	18-20 hours overnight @ 37°C; 30-60 min @ 56°C	Overnight @ 37 °C; 30 min @ 56 °C	Overnight @ 37 °C; 45 min @ 56 °C	37°C overnight (19 ± 1 h), 30-min @ 56°C	Overnight @ 37 °C; 30 min @ 56 °C	18-20 hours overnight @ 37°C; 45 min @ 56°C
RBC source	Horse	Avian, mammalian	Human (stabilized)	Turkey RBC	Avian, mammalian	Guinea pig RBCs
Virus tested	H5N1	General protocol	type A (H1N1, H3N2, H5N1) and type B	type A (H1N1, H3N2)	type A (H1N1, H3N2), and type B	A/Texas/71/2017 (H3N2), A/Singapore/INFIMH-16-0019/2016 (H3N2)
RBC concentration	1%	0.75% (mammalian), 0.5% (avian)	0.08%	0.50%	1.0% (mammalian), 0.75% (avian)	0.75%
Microtitre plate type	U bottom plate	U (mammalian), V (avian) bottom plate	V-bottom	V-bottom	U (mammalian), V (avian) bottom plate	U bottom plate
Assay buffer	PBS	PBS	DPBS + 1% BSA	PBS	PBS	PBS
Assay buffer volume	25 µl	25 µl	25 µl	25 µl	25 µl	25 µl
Final assay volume	100 µl	100 µl	75 µl	100 µl	100 µl	100 µl
Virus antigen (HAU/25 µl)	4	4	4	4	4	4
First incubation (after virus addition)	at least 30 min	15 min	45 min	30 min	30 min	30 min
Second incubation (after RBC addition)	60 min	60 min (mammalian), 30 min (avian)	75 min	30 min	60 min (mammalian), 30 min (avian)	60 min
Assay temperature	Room temperature	Room temperature	Room temperature	Room temperature	Room temperature	Room temperature (22°C +/- 2°C).
Assay plate readout	By eye, tilted	By eye, avian tilted	Microscope (40-fold magnification)	By eye, tilted	By eye, avian tilted	CypherOne instrument
Endpoint determination for seropositivity	Fully precipitated RBC	Fully precipitated RBC	Clear to irregular shaped RBC dot	Fully precipitated RBC	Fully precipitated RBC	Fully precipitated RBC
Reference material	Individual clinical sera	Varies with virus strain and RBC source	NIBSC sheep serum	Pooled in-house reference standards	Individual clinical sera	Pooled in-house reference standard
Parameters tested/assessed	Precision, specificity, linearity, robustness	NA	Precision and accuracy, Linearity and range, robustness, specificity	Precision, reproducibility, specificity, sensitivity, seroprotection.	NA	Matrix effect, precision, accuracy, linearity, range, limits of detection, limits of quantitation, robustness, specificity, sensitivity, seroprevalence
Precision analysis method	No, (%) with indicated result within twofold of GMT	NA	No, n and (%) of samples within twofold titre range	%GCV, (%) with indicated result within twofold of GMT	NA	%GCV
Were statistical analysis methods reported	yes	NA	yes	yes	yes	yes
Which statistical methods and packages were used	regression (correlation coefficient)	NA	linear regression (slope, intercept, correlation coefficient)	chi-square test	linear regression (correlation coefficient)	Correlation analysis, prediction ellipses, percent linearity, linear regression (correlation coefficient, slope, p value, confidence intervals), mixed models, R package ggplot
Software used for statistical analysis	Not reported	NA	Graphpad Prism v5.01	Prism 4 statistics software	Not reported	SAS, R
Analysis formulae reported	Not reported	NA	Not reported	Not reported	Not reported	Reported

Here, we report on the qualification of an HAI assay for two representative H3N2 influenza strains, A/Texas/71/2017 and A/Singapore/INFIMH-16-0019/2016. to support work of CIVICs human influenza challenge study ClinicalTrials.gov Identifier: NCT04978454. A/Singapore/INFIMH-16-0019/2016 chosen to support work of CIVICs influenza vaccine study ClinicalTrials.gov Identifier: NCT04960397. Quantification of antibody titers by the HAI assay is dependent on the process of hemagglutination, or the binding of hemagglutinin glycoproteins on the surface of influenza virus to sialic acid receptors on red blood cells (RBCs). The determination of precise and accurate HAI titers can prove challenging due to the inability of modern H3N2 influenza strains to agglutinate avian RBCs and the acquired ability of these strains to agglutinate RBCs through neuraminidase (NA) activity (13). Here we have leveraged the use of guinea pig RBCs and the inclusion of the neuraminidase inhibitor Oseltamivir to prevent NA-mediated agglutination (13). Qualification of the HAI assays was performed in accordance with the United States Food and Drug Administration (FDA) Guidance for Industry: Bioanalytical Method Validation (May 2018) (14), the International Council for Harmonization [ICH] Tripartite Guideline (15) and guidance from NIAID/DMID, as relevant. System suitability criteria was evaluated to ensure the data was suitable for inclusion in the qualification parameter analysis and can serve an important first step for labs attempting to qualify new strains. Parameters tested for this assay qualification include matrix effect, linearity, precision including intermediate precision, accuracy, range, limits of detection and quantitation, specificity, and robustness. We additionally report here on improved methods of data processing, documentation, traceability, and analysis to ensure efficient and accurate data interpretation.

## Materials and methods

### Influenza strains

A/Texas/71/2017 (H3N2, International Reagent Resource Cat# FR-1622) was propagated in Madin-Darby Canine Kidney cells (MDCK) cells (ATCC, Cat# CCL-34) and used for the initial qualification. A/Singapore/INFIMH-16-0019/2016 (H3N2, International Reagent Resource, Cat# FR-1590) was also propagated in MDCK cells and used in the extended partial qualification. Two lots were tested during robustness analysis.

### Guinea pig red blood cells

Guinea pig RBCs (100%, Innovative Research, Cat# IGPRBC10ML, Lot # 34252-01, 34252-02, 35043, 37615) were prepared at 0.75% in phosphate buffered saline (PBS, pH 7.4 Ca2+ & Mg2+ free, Gibco, Cat # 10010-023) for qualification analysis. Two lots of RBCs were tested during robustness analysis.

### Oseltamivir phosphate

The neuraminidase inhibitor, Oseltamivir (Selleckchem, Cat # S2597, Lot# S259705), was added into the 0.75% guinea pig RBC solution at a final concentration of 20nM. Oseltamivir was also added into the PBS used to dilute the serum samples at a final concentration of 20nM.

### Receptor Destroying Enzyme II

All sera and plasma samples were treated with Receptor Destroying Enzyme type II (RDE II) prior to use in the HAI assay. The lyophilized receptor destroying enzyme II (Hardy Cat # 370013, Lot# 600092, 631082) was reconstituted using phosphate buffered saline (PBS, pH 7.2 Ca2+ & Mg2+ free, Gibco Cat # 20012-027). The samples were treated with RDE II at a 3:1 ratio at 37°C in a heating block for 18 hours. The RDE II was then heat inactivated at 56°C in a heating block for 45 minutes. Following heat inactivation, the RDE II treated samples were diluted with PBS (pH 7.4 Ca2+ & Mg2+ free, Gibco Cat # 10010-023) to bring the samples to a final dilution of 1:10. Diluted samples were aliquoted if needed for multiple tests and stored at -20°C.

### Positive and negative controls

A panel of H3 reactive monoclonal antibodies was evaluated, leading to the selection of Ab2210 IgG1 as the positive control (Lot # 99BMH, 33JWM). Ab2210 IgG1 binds to the apex of the HA protein (16) and has an HAI titer of 640 against A/Texas/71/2017 and 160 against A/Singapore/INFIMH-16-0019/2016 when used at 100 µg/ml starting concentration, (1:10 dilution of 1mg/ml stock). The HIV specific monoclonal antibody 7B2 IgG1 mAb (Lot # 180615PPF) (17) and Anti-West Nile Virus-E protein (WNV-E, Clone MGAWN1 reference lot 1-FIN-1027 humanized IgG1, BEI Resources, Cat # NR-31082, Lot# 61277164) were used as negative assay controls at 10 µg/ml starting concentration, prepared as a 1:10 dilution of 0.1 mg/ml stock. These controls were used in determining the system suitability criteria and throughout the HAI assay qualification experiments.

### Test samples - A/Texas/71/2017 (H3N2) qualification

•Linearity, precision, accuracy, range, LOD, LOQ and robustness testing: There are currently no commercially available reference standards with known HAI titers against influenza A/Texas/71/2017. A small volume of human pooled convalescent serum to A/Texas/71/2017 was provided to Duke University from the Centers for Disease Control and Prevention (CDC). Due to the limited amount of the CDC antiserum, clinical plasma samples from patients vaccinated with the 2019-2020 seasonal influenza vaccine were profiled for HAI titers against A/Texas/71/2017 to create a panel of sixteen samples with a range of antibody levels. Two of these plasma samples with HAI titers ranging from 320 to 1,280 were pooled to serve as an in-house reference standard (IHRS).Serum samples from this vaccinated cohort were not available. All clinical samples were used with IRB approval.
•Matrix effect testing: Normal human serum (Sigma-Aldrich, Cat # H4522, Lot # SLBX6353) and influenza negative human plasma (BioIVT, Cat # HMPLCPD-RPP1, Lot # HMN410054/00002) with HAI titers ≤ 20 were used as the base for matrix effect testing. The normal human serum was also included in specificity testing.
•Specificity testing: World Health Organization (WHO) serum and supplemental antiserum were obtained through the International Reagent Resource (IRR), Influenza Division, WHO Collaborating Center for Surveillance, Epidemiology and Control of Influenza, Centers for Disease Control and Prevention, Atlanta, GA, USA.
  ◦Influenza Normal Control Goat Serum (IRR Cat # FR-1377, Lot # 63461731) and Influenza A(H7N9) Reference Ferret Antiserum (IRR Cat # FR-1250, Lot # 61982458) have HAI titers ≤20 and were considered negative when used during specificity testing.
  ◦The 2014-2015 WHO Antiserum, Influenza A(H3) Reference Goat Antiserum (IRR Cat # FR-1351, Lot # 1415H3AS) and 2019-2020 WHO Antiserum, Influenza A(H3) Reference Goat Antiserum (IRR Cat # FR-1683, Lot # 1920H3AS) have HAI titers ≥ 40 and were considered positive when used during specificity testing.
  •Sensitivity testing: A panel of twenty serum samples purported to have limited or no cross reactivity to influenza A/Texas/71/2017 (provided courtesy of NIH/NIAID Division of Microbiology and Infectious Diseases, DMID 10-0016 ClinicalTrials.gov Identifier: NCT01317745, DMID 05-0130 ClinicalTrials.gov Identifier: NCT00311675) were used for sensitivity testing during qualification.
  •Seroprevalence survey: Ten HIV seronegative human serum samples with unknown influenza status (BioreclamationIVT/BioIVT) were used for a preliminary determination of the seroprevalence of HAI titers against A/Texas/71/2017.

### Test samples - A/Singapore/INFIMH-16-0019/2016 (H3N2) qualification

•Linearity, precision, accuracy, range, LOD and LOQ testing: A panel of twenty human serum samples consisting of 13 unique subject IDs and sample days 8, 36, 57 and 209 with known positive HAI titers (FluGen H3N2-V003 ClinicalTrials.gov Identifier: NCT03999554) was profiled for HAI titers against A/Singapore/INFIMH-16-0019/2016. Five of these serum samples with HAI titers ranging from 640 to 1,280 were pooled to serve as an in-house reference standard (IHRS)
•Specificity testing: World Health Organization (WHO) serum and supplemental antiserum were obtained through the International Reagent Resource (IRR), Influenza Division, WHO Collaborating Center for Surveillance, Epidemiology and Control of Influenza, Centers for Disease Control and Prevention, Atlanta, GA, USA.
  •Influenza A(H3N2)v Reference Ferret Antiserum (IRR Cat # FR-1000, Lot # 60711729); 2019-2020 WHO Antiserum, Influenza A(H1N1)pdm09 Reference Goat Antiserum (IRR Cat # FR-1682, Lot # 1920H1AS); 2018-2019 WHO Antiserum, Influenza B Reference Goat Antiserum, B/Victoria Lineage (IRR Cat # FR-1613, Lot # 1819BVAS); 2019-2020 WHO Antiserum, Influenza B Reference Goat Antiserum, B/Yamagata Lineage (IRR Cat # FR-1685, Lot # 1920BYAS); Influenza A(H7N9) Reference Ferret Antiserum (IRR Cat # FR-1250, Lot # 61982458) have HAI titers ≤10 and were considered negative when used during specificity testing. Normal Goat Serum (MP Biomedicals Cat# 2939149, Lot # S1608) was used as a negative sample in specificity testing.
  •The 2016-2017 WHO Antiserum, Influenza A(H3) Reference Goat Antiserum (IRR Cat # FR-1487, Lot # 1617H3AS); 2017-2018 WHO Antiserum, Influenza A(H3) Reference Goat Antiserum (IRR Cat # FR-1562, Lot # 1718H3AS); 2018-2019 WHO Antiserum, Influenza A(H3) Reference Goat Antiserum (IRR Cat # FR-1612, Lot # 1819H3AS); and 2019-2020 WHO Antiserum, Influenza A(H3) Reference Goat Antiserum (IRR Cat # FR-1683, Lot # 1920H3AS) have HAI titers ≥ 640 and were considered positive when used during specificity testing.

### Hemagglutination inhibition assay

Using an established HAI protocol as the template (18), the HAI assay was performed by adding 25 µl of PBS (pH 7.4) to column 1 and to columns 3-11 of the 96 well U bottom plate for serum dilutions. For the back titration control, 50 µls of PBS was added to all wells of the row. A red blood cell control was included in column 12, 50 µl of PBS was added to this column. The RBC control contained only RBCs without sample or virus. A serum control was included in column 1, 25 µl of diluted RDE treated serum samples were added to this column to monitor non-specific agglutination in the individual serum samples. To perform serum dilutions, 50 µl of diluted RDE treated serum samples were added to column 2 of the plate. 50 µl of the positive and negative controls were also added into column 2 of their respective control rows. Two-fold serial dilutions were performed, discarding pipet tips after each mixing step. The dilution series was continued from column 3 to column 11, discarding the remaining 25 µl from column 11. The influenza virus was removed from the -80°C freezer and thawed at room temperature immediately before use. The final HA unit of the virus was adjusted to 8 HA units with PBS (pH 7.4) and 25 µl of diluted virus was added to columns 2 - 11, except for the back titration control row. To perform the back titration control, 50 µls of stock virus was added into column 2 of the back titration control row and mixed several times to perform an initial 1:2 dilution. Two-fold serial dilutions of the back titration control row were performed. Plates were tapped gently to mix the serum and virus then incubated for 30 minutes at room temperature. Immediately prior to adding RBCs, they were inverted several times to ensure cells were fully resuspended and 50 µl of diluted RBCs were added to all wells of the plate and incubated for 60 minutes at room temperature to allow RBCs to precipitate. All material in contact with influenza virus stocks was decontaminated with freshly prepared 10% bleach. This includes all vials, tubes, reservoirs, and pipet tips. To determine antibody titers, plates were scored for the presence of hemagglutination using the CypherOne HAI plate reader [InDevR, software version 4.0.0.19 (19)]. The CypherOne software was used to build a plate template to document the location of assay controls position of test samples and the orientation of the dilution series within the plates. A plate list was used to document the specific location of samples and details of the dilution series and to standardize the data analysis parameters used to make the titer determinations including the instrument calibration factor and transition point applied across all plates within an assay. The antibody titer corresponded to the inverse of the serum dilution of the last well that contained an RBC precipitate. Results were exported as both CSV files and annotated images. These files were saved in a secure network drive for data processing and analysis. Geometric mean titers (GMTs) were determined for each set of sample replicates either within an individual assay plate or across multiple plates within an assay (depending on the experimental design). Although the individual replicate titers values can only be the inverse of a value in the dilution series, GMT values other than the inverse of a dilution can occur due to the allowable two-fold variation between duplicates. This occurs when one replicate has a titer value one dilution higher or lower than the other replicate.

### Qualification parameters, study designs and acceptance criteria

An assay qualification plan including recommended acceptance criteria was prepared and approved before the conduction of qualification experiments.

#### System suitability criteria

The system suitability criteria were established based on the performance of the positive control, negative control, red blood cell control and back titration controls. Red blood cell controls were included on each assay plate. One plate within the assay contained the positive control, negative control and back titration controls. For acceptance, the positive control titer must fall within 2-fold of the expected titer value, and the negative control must have a titer<20. The red blood cell controls must all be fully precipitated. The back titration control titer must be within 2-fold of the expected titer value. If any of these criteria were not met, the assay was to be considered as failed and a repeat was performed.

#### Matrix effect

Due to the lack of matched serum and plasma pairs, matrix effect was evaluated by spiking in the positive control antibody Ab2210 IgG1 into RDE II treated normal human serum and negative human plasma samples diluted 1:20 in PBS, both with a HAI titer against A/Texas/71/2017 ≤ 20. Ab2210 IgG1 was diluted 2-fold beginning at a concentration of 400 µg/ml to match the dilution series performed in PBS for assay linearity testing. Each assay plate was tested in duplicate by two scientists for a sample size of 4 replicates, two per matrix type, within each of the two assays. Four replicates for each of the eight titers in the dilution series, from two assays, resulted in 64 replicate data points, 32 GMTs, 16 for each sample type, which were used in correlation analysis. Matrix effect was determined by comparing the titer values obtained with Ab2210 IgG1 spiked into negative serum versus antibody titers obtained with Ab2210 IgG1 spiked into negative plasma and demonstrating correlation with expected correlation coefficients ≥ 0.9. The percent linearity for each antibody dilution in plasma or serum was also be calculated to aid in the determination of assay linearity. Acceptable dilutional percent linearity was defined as dilution corrected antibody titers that varied no more than 50% to 200% between doubling dilutions. Values had to be 50% to 200% to allow for 2-fold variability in titer values.

#### Precision and accuracy

The precision of the assay was determined by performing dilutions of the pooled plasma IHRS at 1:20, 1:80, 1:320 and 1:640 in PBS to create samples with high, medium, low, and near LLOQ response levels. The exact dilution scheme was determined from results of the linearity testing with the very low response dilution corresponding to the last dilution to retain acceptable percent linearity. The two-fold serial dilutions were starting at a 1:20 dilution and continued to a 1:5120 dilution. The corresponding titer of each of these antibody dilutions was determined. To evaluate intra-assay repeatability these dilution series were performed in duplicate on a plate with a single scientist testing 5 assay plates on day one for a total of 10 replicates. To evaluate intermediate, inter-assay precision, the above assay was performed with a second scientist testing two additional plates, increasing the total replicate count to 14. Intermediate precision continued to be assessed by repeating the assay on a second day with two scientists testing two plates each containing duplicate dilution series. This resulted in additional 8 replicates to the existing 14 replicates for a final total of 22 replicate titer values, which yielded 11 GMTs, per dilution. Mixed models’ analysis was used to calculate the %GCV for intermediate precision and repeatability, by dilution. The recommended acceptance criterion for the evaluation of repeatability and intermediate precision (% CV) for high, medium, and low response levels was ≤ 20%, and for the near LLOQ level was ≤ 25%. Relative accuracy (mean bias) was calculated for each of the four levels of testing. It was expected that the relative error (%RE) for high, medium, and low response levels would be ≤ 20%. The expected relative error (%RE) for the near LLOQ value was expected to be ≤ 25%. For accuracy, the acceptable level of variability in the assay was 2-fold variation in titer values, so values of 50% to 200% to allow for 2-fold variability in titer values were also acceptable.

#### Assay linearity

Assay linearity was determined by performing a two-fold dilution of the pooled plasma IHRS for 8 serial dilutions from 1:10 - 1:1280 and determining the corresponding GMT value. Two scientists contributed to the linearity analysis and generated data for two replicate curves in each assay. Each assay plate was tested in duplicate by two scientists for 4 replicates. GMT was calculated by dilution, within assay replicates. Two replicates over eight dilutions yielded 16 GMTs. Linearity was first evaluated by visually assessing the titer versus antibody dilution in the CypherOne instrument graphics. Linearity was also evaluated through regression analysis and plots of titer versus sample dilution. Linearity results were described by correlation coefficient (R), slope, 95% confidence interval of the slope of the least squares regression line, and the coefficient of determination (R^2^). The expected coefficient of determination, R^2^ was ≥ 0.9. The percent linearity for each sample dilution was also calculated. Acceptable percent linearity was defined as dilution corrected antibody titers that varied no more than 50% to 200% between doubling dilutions. Values had to be between 50% to 200% to allow for 2-fold variability in titer values. Assay linearity was also evaluated through regression analysis, as described above, of plots of titer versus sample dilution obtained during matrix effect evaluation.

#### Range, limits of detection and quantitation

Range, LOD and LOQ were determined by evaluating the sample dilutions with acceptable assay linearity, as well as precision and accuracy, from data generated in the assay linearity experiments. Antibody dilutions that lacked acceptable percent linearity, precision and accuracy were used to inform the range of the assay and the limits of detection and quantitation. The limit of detection was pre-defined as<10 based on the lowest titer tested in the linearity experiments.

#### Robustness

The robustness of the assay was demonstrated by evaluating deviations in incubation times and temperatures as well as the impact of changing lots of red blood cells and virus stock. Robustness testing was conducted by performing dilutions of the pooled plasma IHRS at 1:20, 1:80, 1:320 and 1:640 in PBS to create samples with high, medium, low, and very low (near LLOQ) response levels. The exact dilution scheme was determined from results of the linearity testing with the very low response dilution corresponding to the last dilution to retain acceptable percent linearity. These dilutions were performed in duplicate on 6 plates. To assess the impact of variations in erythrocyte incubation length on assay robustness, each of three plates containing the pooled plasma IHRS dilution series, as described above, were incubated for either 45 minutes, 1 hour or 1 hour and 15 minutes during the red blood cell incubation. These three incubations were performed at room temperature (22°C +/- 2°C). To assess the impact of temperature fluctuations during the assay, a fourth assay plate was tested using red blood cells at 4°C that were not equilibrated to room temperature prior to use. This red blood cell incubation was performed for the standard 1-hour timeframe. To assess the impact of changing reagent lots, a fifth and sixth assay plate was tested using a new lot of red blood cells and a new lot of A/Texas/71/2017, respectively. These plates were tested using standard incubation times and temperatures. The conditions were tested independently on the same day but with use of shared prepared reagents when available and appropriate. The corresponding titer of each of these antibody dilutions at each of these conditions was determined and precision and accuracy analysis were performed to calculate the % GCV and % relative error. To be considered robust, each assay condition tested was expected to retain precision and accuracy when compared to the standard assay conditions. The expected values for acceptable precision (% GCV), and accuracy (% RE), for high, medium, and low response levels was ≤ 20%, and for very low/near – LLOQ level, ≤ 25%.

#### Specificity

The specificity of the assay was evaluated by testing the CDC pooled convalescent serum specific to the A/Texas/71/2017 influenza strain and H3 reference goat antiserum, along with a panel of normal serum from different species and ferret antiserum to a heterologous strain. This experiment was performed on two separate occasions using first a 1:20 starting dilution and then a 1:10 starting dilution of serum samples. Each assay plate was tested in duplicate by two scientists for a total of 4 replicates. Acceptable assay specificity was determined by the ability of the assay to correctly identify three homologous strains and three heterologous strains. A titer of ≥1:40 was considered positive.

#### Sensitivity and seroprevalence

Sensitivity is a measure of the ability of the assay to detect titers near the lower limits of detection and quantitation. The seroprevalence of an antigen is the frequency that an antibody response to that antigen is detected within a population. Thirty samples were tested by each scientist, and percentage of samples below the LOD, were assessed. There were no pre-specified acceptance criteria for these parameters.

#### Extended qualification

To eliminate the need to fully re-qualify the HAI assay for each new strain of virus in evaluation, the original qualification using the A/Texas/71/2017 strain was extended to qualify the H3N2 influenza strain A/Singapore/INFIMH-16-0019/2016. An extended partial qualification is performed when an assay has previously been fully qualified for a similar antigen. This extended partial qualification evaluates assay parameters that have the potential to be impacted when a new antigen is used in the assay and will rely on the results obtained in the original assay qualification for parameters that are not expected to be impacted by the addition of a new antigen. The partial qualification utilized the same critical assay reagents and controls as those used in the original qualification for the HAI assay. The extended qualification served a dual purpose. Firstly, and most importantly, it helped to evaluate if the assay design and analysis methods can be effortlessly transferred in testing and qualification of another virus strain. Secondly, the extended qualification was used as an opportunity to improve upon any potential gaps in study design that were identified during the initial qualification analysis and to develop a custom data pipeline for HAI data processing. The parameters tested for the extended qualification include system suitability criteria, precision, accuracy, linearity, range, LOD, LOQ, and specificity. During the original qualification analysis, eight two-fold dilutions of the IHRS beginning at a 1:10 dilution were used to generate a dataset for linearity, which was in turn used for range, LOD and LOQ determination. Precision and accuracy were confirmed on this linearity dataset again to determine range, apart from the original dataset generated for precision. For the extended qualification, rather than generating datasets by parameter being tested, when the same sample (titrated IHRS) was used for data generation, linearity, precision, accuracy, range, LOD and LLOQ, were all determined using one dataset. Eight, two-fold dilutions of the IHRS beginning at a 1:10 dilution, were generated, and the corresponding GMTs were calculated. Each assay tested duplicate sample dilutions on duplicate plates resulting in 4 individual replicates and 2 GMT values, from each assay. The assay was performed by two scientists, and each scientist generated data from three assays, to provide 12 GMTs and 24 individual replicates in total. One set of curves failed quality control criteria due to experimental error, and was excluded from analysis, resulting in 11 GMTs, at each of the eight dilutions, and a total of 88 observations for final analysis (**Supplementary Table 5**). This improvement helped generate a more balanced dataset, with the same number of replicates at all eight dilutions. Also, this increased the number of observations used for statistical analysis and helped avoid repeated % GCV and % RE analysis on a separate dataset when determining range. Thus, the additional datapoints enabled the more efficient analysis of linearity and range and in turn added more variance to the models. Furthermore, we also developed a standardized data pipeline and custom HAI Module hosted on LabKey (20) infrastructure using the test files generated during qualification. The data processed through this portal were used during extended qualification to ensure data integrity and automation of processing and tracking of controls.

### Quality control and data processing

For qualification of the H3N2 influenza strain A/Texas/71/2017, raw data were exported from the CypherOne (19) software as csv files using the standardized template. A standardized data pipeline was developed for HAI assay. Raw data generated by the CypherOne are passed through a series of processing steps within this pipeline. These processing steps are highly structured to ensure adherence to assay protocols and expectations of the data quality and integrity. A custom HAI Module was developed in house for compiling all data pipeline steps and executing them in an internal database, termed the Portal database [LabKey Server, software version 21.3 (20)]. R programming language was used for scripting and incorporating processing in infrastructure provided by LabKey (20). The HAI Module automated data processing steps and aggregated data in a standard format in one database, to document any variations in the data generation that resulted from multiple scientists performing the assay and minimizing potential human error. Data was stored on the Portal to prevent any manipulations or modifications to the original assay data, regardless of the access level, and logged history of any action performed. A system of error and warning messages was designed as part of this data pipeline to communicate any irregularities with the data that should be addressed by the operator prior to data advancing in the pipeline. Metadata was provided by the operator during initial upload steps to the Portal in a highly restricted and structured manner and was parsed and assigned to the raw data. This allowed data to be stored in standardized format without any data points or associated metadata missing. As part of the processing, summary data was generated: geometric mean and %CV of each replicate titer value was calculated to show variability across replicates.

A Quality Control (QC) process was incorporated in the data pipeline and scripted to execute automatically during data upload to the Portal. Quality of the data was determined using the following criteria: RBCs were expected to be fully precipitated (well value measurements above 1000), serum control had no detected non-specific agglutination (well value measurements above 1000), back titration titer value was within two-fold of documented virus titer value, positive control titer value was within two-fold of documented positive control titer value, negative control wells were fully agglutinated (well value measurements below 1000). These QC flags were aggregated into reports that facilitated streamlined data review. Additional QC metrics were applied as part of the data quality and integrity check, which allowed data review on a summary level and tracked historical performance. These additional QC metrics included checks that all titer values were of base 10, replicate titer values were within acceptable range of variability (two-fold for duplicates, four-fold for triplicates, etc.) and back titration titer value is of expected format. Historical performance for positive controls, negative controls, and back titration were tracked and viewed as non-editable graphs, which allowed performance review of the controls on specific study or virus levels.

**Figure 1:**
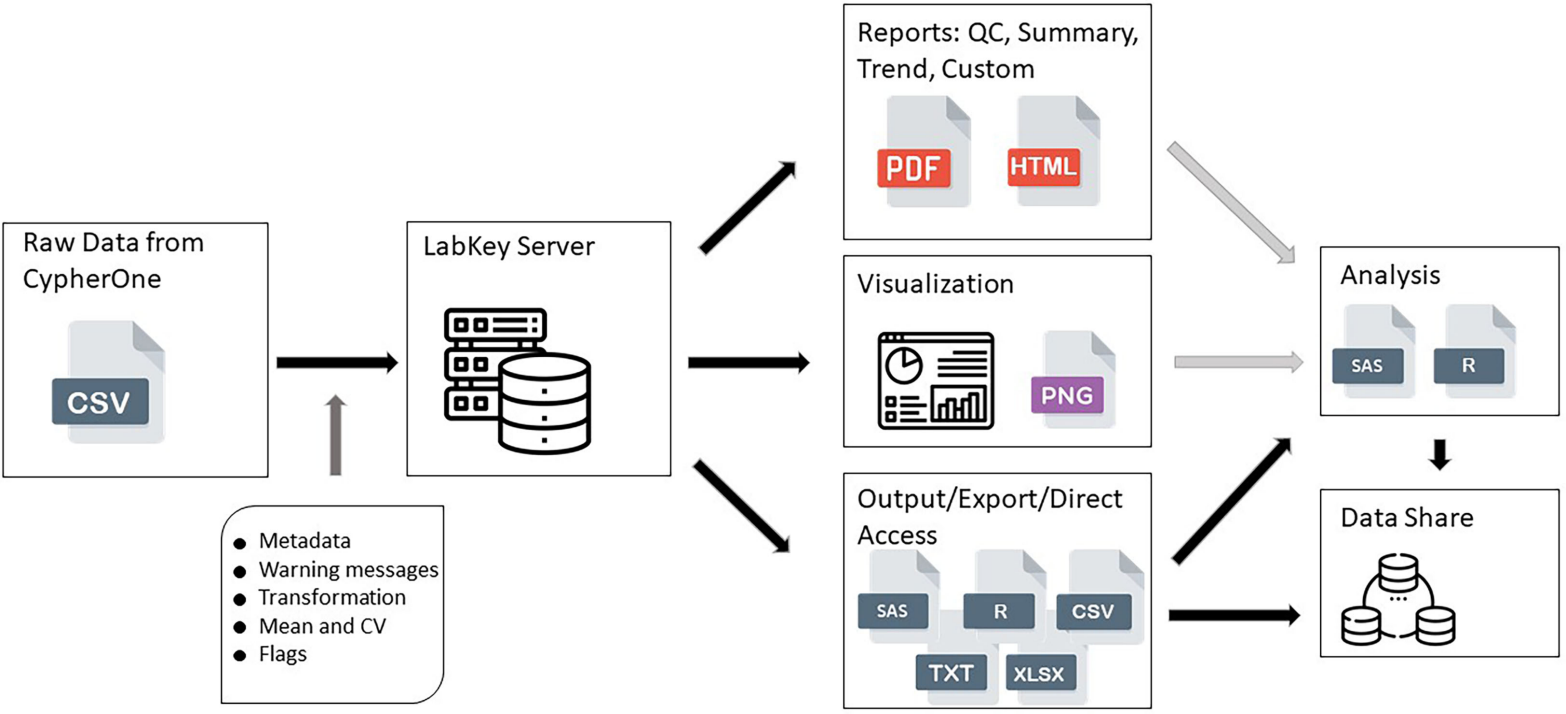
HAI assay design data pipeline. This figure shows the data flow from raw data generated by CypherOne Software to processed data used for analysis and data sharing. Light grey arrows indicate decision making steps: generated reports and visualization assists with determining the quality of the data and analysis readiness. Dark grey arrow indicates main processing steps performed during data upload. Black arrows indicate data pipeline steps which are scripted and secured.

All raw data, processed data, and quality control data were available for view and access after successful upload onto the Portal (**Figure 1**). All reports, views, and graphs that are part of this HAI Module could be generated any time after data becomes available, which ensures flexibility to access data and its supplemental materials from one database where all information is linked together. All data pipeline steps are stored in the background of the server which requires a specific access level and have version control implemented, which makes this data pipeline secure and highly structured, preventing any unauthorized changes or updates to any of its steps or components.

Data processing, analysis and plot of data was generated using Statistical Analysis Software (SAS) and R statistical software. R version 4.2.2 (2022) The R Foundation for Statistical Computing, Platform: x86_64-w64-mingw32/x64 (64-bit), ggplot package, was used for generating certain plots. Statistical analysis was performed using SAS (r) Proprietary Software 9.4 (TS1M7; Copyright (c) 2016 by SAS Institute Inc., Cary, NC, USA), licensed to DUKE UNIVERSITY - T&R - SFA - NCICU GRANT, Site 70082794. Development of the standardized data pipeline was initiated with qualification experiments for H3N2 influenza strain A/Texas/71/2017 and fully implemented for extended qualification of the H3N2 influenza strain A/Singapore/INFIMH-16-0019/2016.

### Analysis

Titers of<20 or<10 dilution were converted to half of the lowest dilution tested in assay, in this case 10 or 5, respectively, to enable statistical analysis. GMTs were determined for each set of sample replicates within assay or assay plate, as applicable, and log 10 transformed for statistical analysis. These GMTs were used to determine the %GCV and relative error. The formulas used are included below:


$$Standard\;Deviation\;Intermediate\;Precision=\sqrt{Intra\;assay\;Variance\;+Inter\;assay\;Variance}$$

$$\%Geometric\;Coefficient\;of\;Variation=\;\sqrt{e^{\left({\left[SD_{log10}\times \ln\left(10\right)\right]}^{2}\right)}-1}\;\times 100$$

= standard deviation (SD) of log10 transformed data. $Here\;SD_{log10}$

$$\%Relative\;Error=\left(\frac{mean\;observed\;value-expected\;value}{expected\;value}\right)\times 100$$

$$\%Relative\;Error=\left(10^{\left(mean\;log10\;observed\;value\;-\;log10\;expected\;value\right)}-1\right)\times 100$$

$$\%\;Linearity=\;\frac{\left(Titer\;\times Dilution\;factor\right)}{\left(Previous\;Titer\;\times Previous\;Dilution\;factor\;\right)}\;\times 100$$

SAS GEOMEAN function was used for calculation of GMTs. For linear regression analysis, for linearity and matrix effect testing, SAS PROC GLM was used, and the results were confirmed using PROC REG. Model statements were log10_Geomean_titer = log10_dilution; log10_Geomean_titer = log10_expected_titer, for the linear regression analysis, as applicable. PROC CORR was used for correlation analysis for matrix effect data. Wherever needed, the 95% confidence intervals were calculated using options SOLUTION and CLPARM, in model statement of PROC GLM. An alpha of 0.05 was used to generate the confidence intervals. Mixed model analysis was used to assess precision on log10 GMTs. The sum of within and between assay variance was used to calculate the %GCV, for precision. PROC MIXED with a random effects model was used to calculate %GCV IP [random effect used in model was assay id]; and %CV intra-operator repeatability [random effect used in model was plate_number], to model the log 10 GMT, as applicable by study design. When there is no variance in this dataset, for cases when all GMTs were the same number, the program would give an error ‘An infinite likelihood is assumed in iteration 0 because of a nonpositive residual variance estimate.’ %CV was set to 0% for such cases since all titer values were the same.

## Results

**Figure 2:**
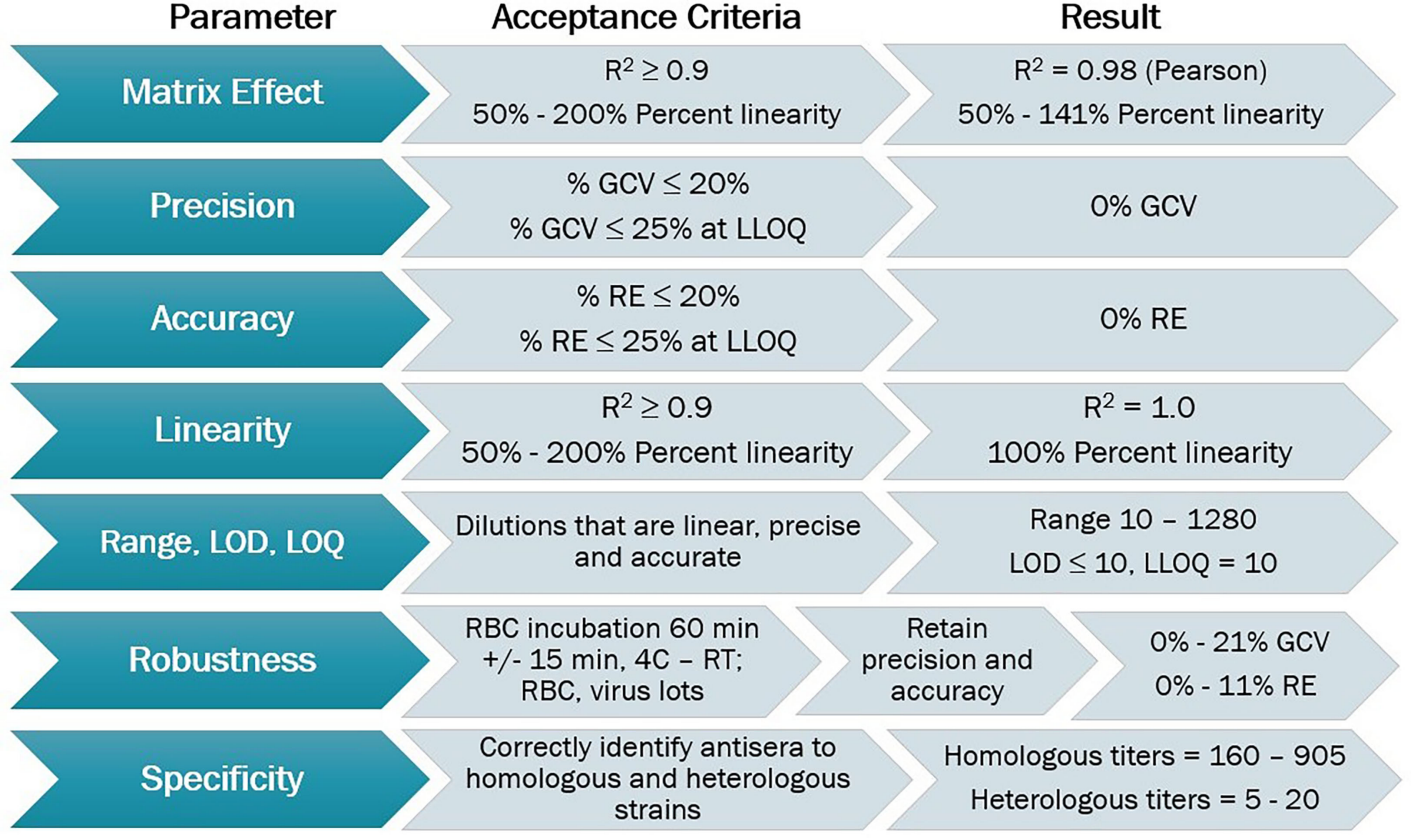
HAI assay qualification for H3N2 influenza strain A/Texas/71/2017, overview of qualification plan and parameters tested during qualification, recommended acceptance criteria, and results obtained.

The HAI assay was qualified using predefined acceptance criteria for each parameter evaluated as a set of metrics that determined the success of the qualification process (**Figure 2**). Parameters tested for the A/Texas/71/2017 HAI assay qualification included matrix effect, precision, accuracy, linearity, range, limits of detection and quantitation, robustness, specificity, sensitivity, and seroprevalence.

### System suitability criteria

**Figure 3:**
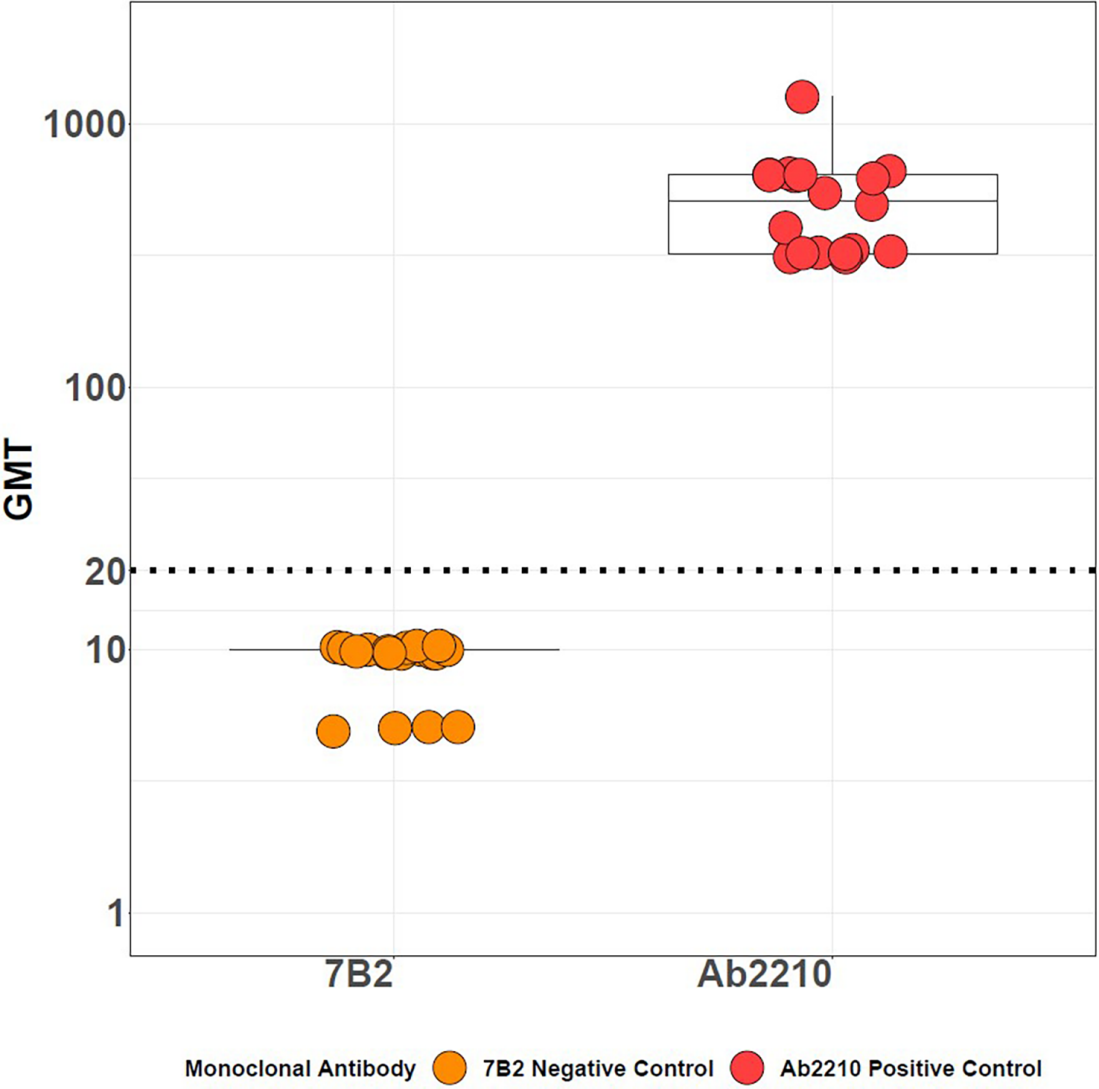
HAI assay qualification on H3N2 influenza strain A/Texas/71/2017, system suitability criteria results. Geometric mean titers [GMTs] for positive control Ab2210 IgG1 and negative control 7B2 IgG1 from all experiments. Nineteen data points plotted for both controls. Titers of<20 or<10 are converted to half of the lowest dilution tested in assay, in this case 10 or 5, respectively, to enable statistical analysis. The Ab2210 IgG1 titers are illustrated with red dots, the 7B2 titers are illustrated with orange dots. The dotted line indicates a GMT of 20.

The SSC were designed to ensure that the assay was performing optimally during each run. All assay controls performed as expected within the acceptable ranges for titer values as described in the methods section. The HAI titers for the positive control Ab2210 IgG1 against the A/Texas/71/2017 strain were within the range of 320 -1280 for all plates tested. The HAI titers for the negative control, 7B2 IgG1, were<20 on all assay plates beginning at a 1:20 dilution and<10 on all assay plates beginning at a 1:10 dilution. The red blood cells controls were all fully precipitated. The back titration control HA titers were within the acceptable range on all assay plates tested. No plates failed due to assay results outside of the limits of the system suitability criteria. Results from the positive and negative control titers across all experiments are plotted in **Figure 3**.

### Matrix effect

**Figure 4:**
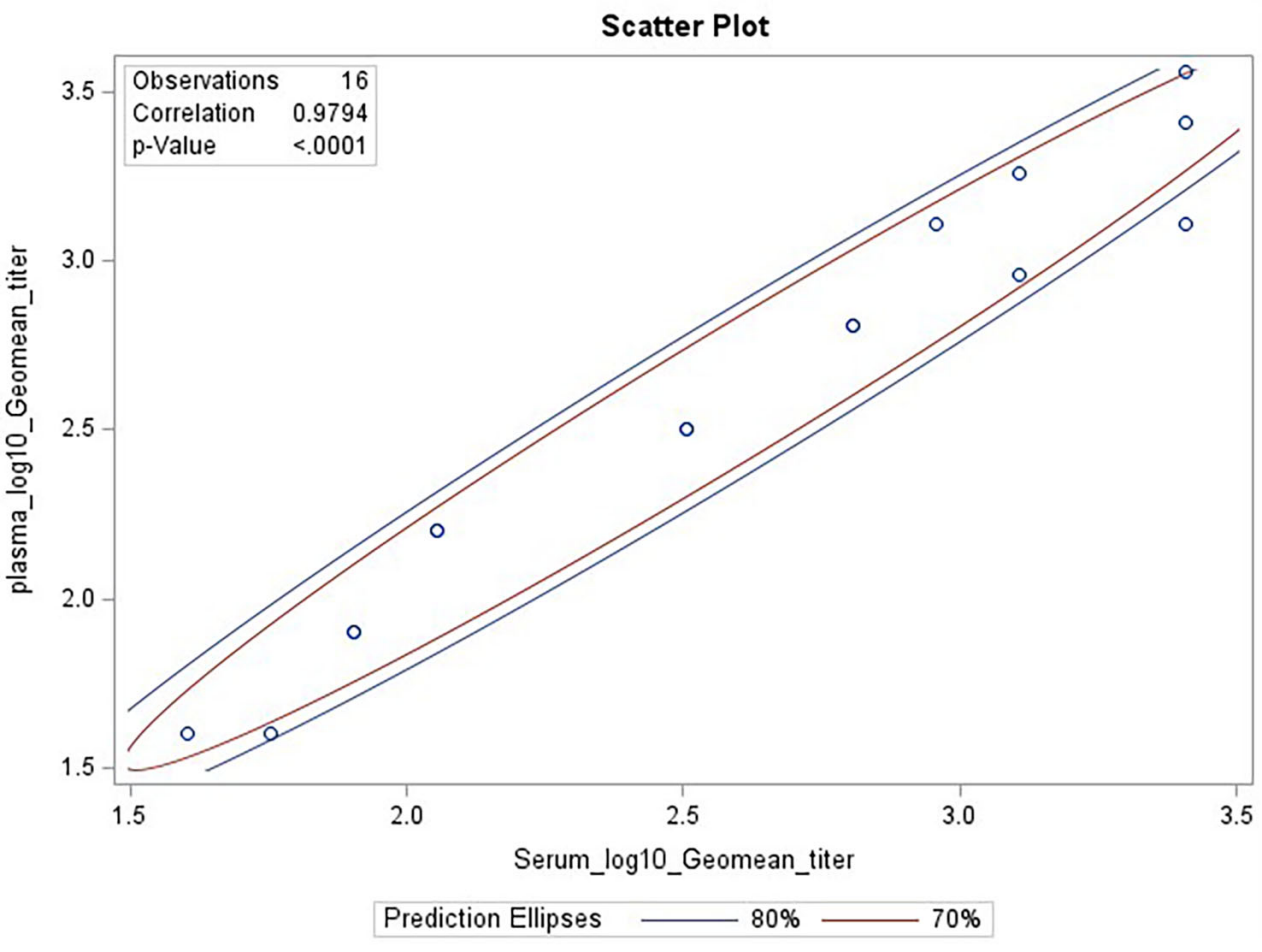
HAI assay qualification on H3N2 influenza strain A/Texas/71/2017, matrix effect testing, linearity analysis results. Scatter plot showing correlation analysis output from SAS CORR procedure, between log 10 transformed GMTs for Ab2210 IgG1 diluted in plasma [plotted on y-axis] and Ab2210 IgG1 diluted in serum [plotted on x-axis]. The 70% and 80% prediction ellipses are plotted in red and blue colors, respectively. Sixteen data points are plotted as circles, some circles overlap due to having same values for both x and y axis. The Pearson correlation coefficient between serum and plasma log10 GMTs was calculated and determined to be 0.98, with a p-value<0.0001.

Biofluids may contain substances that can interfere with the detection of analytes in immunoassays. These are deemed matrix effects and may result in an altered (reduced or increased) expected output signal due to interference of components within the biofluid matrix. GMTs for Ab2210 IgG1 diluted in plasma and Ab2210 IgG1 diluted in serum were calculated for all replicates within the same assays. The resulting 32 geometric mean values, 16 for each sample type, were log 10 transformed. The Pearson correlation coefficient between serum and plasma log10 GMTs was calculated and determined to be 0.98, with a p-value<0.0001. The Spearman correlation was also calculated due to the small sample size. Pearson and Spearman correlations both gave similar R^2^ values, with a Spearman correlation coefficient of 0.97 (**Figure 4**). The scatter plots of serum and plasma titers, along with 80 and 70% prediction ellipses, are shown in **Figure 4**. The percent linearity of the assay in both serum and plasma was maintained with acceptable percent linearity values in the range 50-200% for both sample types, as shown in **Table 2**. There were no fold shifts detected for GMT values in either sample matrix.

**Table 2:** HAI assay qualification on H3N2 influenza strain A/Texas/71/2017, matrix effect testing, percent linearity results.

Obs	Sample Type	Assay	Dilution	GMT	Previous Dilution	Previous dilution GMT	Percent Linearity
1	Plasma	ME_Assay1	1:20	1280	1:10	2560	100
2	Plasma	ME_Assay1	1:40	905	1:20	1280	141
3	Plasma	ME_Assay1	1:80	640	1:40	905	141
4	Plasma	ME_Assay1	1:160	320	1:80	640	100
5	Plasma	ME_Assay1	1:320	160	1:160	320	100
6	Plasma	ME_Assay1	1:640	80	1:320	160	100
7	Plasma	ME_Assay1	1:1280	40	1:640	80	100
8	Plasma	ME_Assay2	1:20	1810	1:10	3620	100
9	Plasma	ME_Assay2	1:40	1280	1:20	1810	141
10	Plasma	ME_Assay2	1:80	640	1:40	1280	100
11	Plasma	ME_Assay2	1:160	320	1:80	640	100
12	Plasma	ME_Assay2	1:320	160	1:160	320	100
13	Plasma	ME_Assay2	1:640	80	1:320	160	100
14	Plasma	ME_Assay2	1:1280	40	1:640	80	100
15	Serum	ME_Assay1	1:20	2560	1:10	2560	200
16	Serum	ME_Assay1	1:40	1280	1:20	2560	100
17	Serum	ME_Assay1	1:80	640	1:40	1280	100
18	Serum	ME_Assay1	1:160	320	1:80	640	100
19	Serum	ME_Assay1	1:320	113	1:160	320	71
20	Serum	ME_Assay1	1:640	80	1:320	113	141
21	Serum	ME_Assay1	1:1280	56.6	1:640	80	141
22	Serum	ME_Assay2	1:20	1280	1:10	2560	100
23	Serum	ME_Assay2	1:40	905	1:20	1280	141
24	Serum	ME_Assay2	1:80	640	1:40	905	141
25	Serum	ME_Assay2	1:160	320	1:80	640	100
26	Serum	ME_Assay2	1:320	113	1:160	320	71
27	Serum	ME_Assay2	1:640	80	1:320	113	141
28	Serum	ME_Assay2	1:1280	40	1:640	80	100

The table depicts the sample GMTs for Ab2210 diluted in plasma and serum [sample type column helps identify sample type], the corresponding assay identifier, dilution, GMT at respective dilution, previous dilution, and GMT at previous dilution. The percent linearity is expected to be in the range of 50 and 200 percent. All observations met this criterion.

### Precision and accuracy

The precision of an assay describes the closeness of agreement between a set of measurements. Intermediate precision expresses the variation observed over multiple days with different scientists and equipment (inter-assay). The %GCVs for repeatability (%GCV intra-assay repeatability) and %GCVs for intermediate precision (%GCV IP) were determined for each dilution level separately. Calculation of %GCV IP was based on the sum of intra-assay and inter-assay variance at each level. A mixed model analysis was used to calculate within and between assay variance for each dilution level. The square root of the sum of within and between run variance was used to calculate the standard deviation for %GCV IP. The square root of the within assay variance was used to calculate the standard deviation for %GCV for intra-assay repeatability. The resulting standard deviations were used to calculate the respective %GCV. The %GCV for intra-operator (intra-assay repeatability) was 0% at all dilutions, as all titers were the same value between all replicates across all plates, as shown in **Table 3A**. The %GCV for intermediate precision was also 0% at all dilutions, as all titers were the same value between all replicates across all plates, as shown in **Table 3B**. All dilutions met acceptable criteria for %GCV for intra-operator repeatability and intermediate precision.

**Table 3A:** HAI assay qualification on H3N2 influenza strain A/Texas/71/2017, precision testing, results for repeatability.

Obs	Dilution	Pr_Assay1_P1	Pr_Assay1_P2	Pr_Assay1_P3	Pr_Assay1_P4	Pr_Assay1_P5	%GCV
1	1:20	640	640	640	640	640	0
2	1:80	160	160	160	160	160	0
3	1:320	40	40	40	40	40	0
4	1:640	20	20	20	20	20	0

The table shows GMTs from within an assay, by dilution, across 5 plates tested in an assay, and the corresponding %GCV for Intra-operator repeatability.

**Table 3B:** HAI assay qualification on H3N2 influenza strain A/Texas/71/2017, precision testing, results for intermediate precision.

Obs	Dilution	Pr Assay1	Pr Assay1	Pr Assay1	Pr Assay1	Pr Assay1	Pr Assay2	Pr Assay2	Pr Assay3	Pr Assay3	Pr Assay4	Pr Assay4	%GCV
		P1	P2	P3	P4	P5	P1	P2	P1	P2	P1	P2	
1	1:20	640	640	640	640	640	640	640	640	640	640	640	0
2	1:80	160	160	160	160	160	160	160	160	160	160	160	0
3	1:320	40	40	40	40	40	40	40	40	40	40	40	0
4	1:640	20	20	20	20	20	20	20	20	20	20	20	0

The table shows GMTs across four assays, by assay and plate identifiers, two scientists performed two assays each. The corresponding %GCV for intermediate precision by dilution is shown in the last column

Accuracy is the agreement between a calculated assay value and an established or predicted value for a tested sample. Relative accuracy (mean bias) was calculated for each of the four levels of testing as described in the precision experiment in the methods section. Relative error (%RE) was calculated as the distance between the average measured value and the expected value. The %RE was 0% at all dilutions, and all mean observed GMTs were the same as the expected GMTs. All dilutions met acceptable criteria for %RE, and the results are shown in **Table 4**.

**Table 4:** HAI assay qualification on H3N2 influenza strain A/Texas/71/2017, accuracy testing, % RE results.

Obs	Dilution	Expected GMT	Mean Observed GMT	%Relative Error
1	1:20	640	640	0
2	1:80	160	160	0
3	1:320	40	40	0
4	1:640	20	20	0

The table shows the expected and observed GMTs, and the corresponding % Relative Error.

### Assay linearity

**Figure 5:**
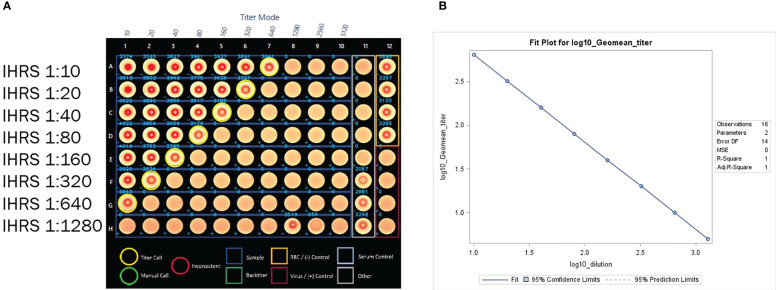
**(A)** HAI assay qualification on H3N2 influenza strain A/Texas/71/2017, linearity testing plate visual. A CypherOne instrument graphic demonstrating assay linearity, from a representative assay with the in-house reference standard (IHRS). The HAI titer decreased two-fold for each two-fold decrease in analyte concentration. **(B)** HAI Assay Qualification on H3N2 influenza strain A/Texas/71/2017, linearity testing results. The plot shows SAS output from REG procedure, showing the linear regression analysis between log10 GMT [plotted on y-axis] and log10 dilution [plotted on x-axis]. The resulting R^2^ and R was 1.

The linearity of an analytical procedure is its ability to provide test results that are directly proportional to the concentration of an analyte in the sample. In the HAI assay, the HAI titer should decrease two-fold for each two-fold decrease in analyte concentration (**Figure 5A**). GMTs were calculated for all replicates within the same assay. Dilutions and the resulting geometric mean titer values were log10 transformed. Linear regression analysis was performed between log10 dilution and log10 geometric mean titer. The resulting R^2^ and R was 1 (**Figure 5B**). The slope was -1, with a 95% confidence interval (-1, -1) and p-value<0.0001. Linearity passed with acceptable R^2^. The percent linearity was 100% for all combinations and showed acceptable percent linearity values between 50-200% (**Table 5**).

**Table 5:** HAI assay qualification on H3N2 influenza strain A/Texas/71/2017, linearity testing, percent linearity results.

Obs	Assay	Dilution	GMT	Previous Dilution	Previous dilution GMT	Percent Linearity
1	Lin_Assay1	1:20	320	1:10	640	100
2	Lin_Assay1	1:40	160	1:20	320	100
3	Lin_Assay1	1:80	80	1:40	160	100
4	Lin_Assay1	1:160	40	1:80	80	100
5	Lin_Assay1	1:320	20	1:160	40	100
6	Lin_Assay1	1:640	10	1:320	20	100
7	Lin_Assay1	1:1280	5	1:640	10	100
8	Lin_Assay2	1:20	320	1:10	640	100
9	Lin_Assay2	1:40	160	1:20	320	100
10	Lin_Assay2	1:80	80	1:40	160	100
11	Lin_Assay2	1:160	40	1:80	80	100
12	Lin_Assay2	1:320	20	1:160	40	100
13	Lin_Assay2	1:640	10	1:320	20	100
14	Lin_Assay2	1:1280	5	1:640	10	100

The table depicts the data for pooled plasma in-house reference standard (IHRS), by assay, dilution, GMT at given dilution, previous dilution, and its GMT at previous dilution. The percent linearity is expected to be within the range of 50 and 200 percent. All observations met the criteria

### Range, limits of detection and quantitation

Range is the interval between the upper and lower concentration of an analyte in the sample for which it has been demonstrated that the analytical procedure has a suitable level of precision, accuracy, and linearity. Range was determined by evaluating the sample dilutions with acceptable assay linearity, precision, and accuracy, using the linearity experimental data. The limits of detection are the upper and lower analyte concentrations that can be detected in the assay but may not be quantified with an acceptable level of linearity, precision, or accuracy. The limits of quantitation are the highest and lowest concentrations of analyte that can be measured with acceptable precision and accuracy.

**Figure 6:**
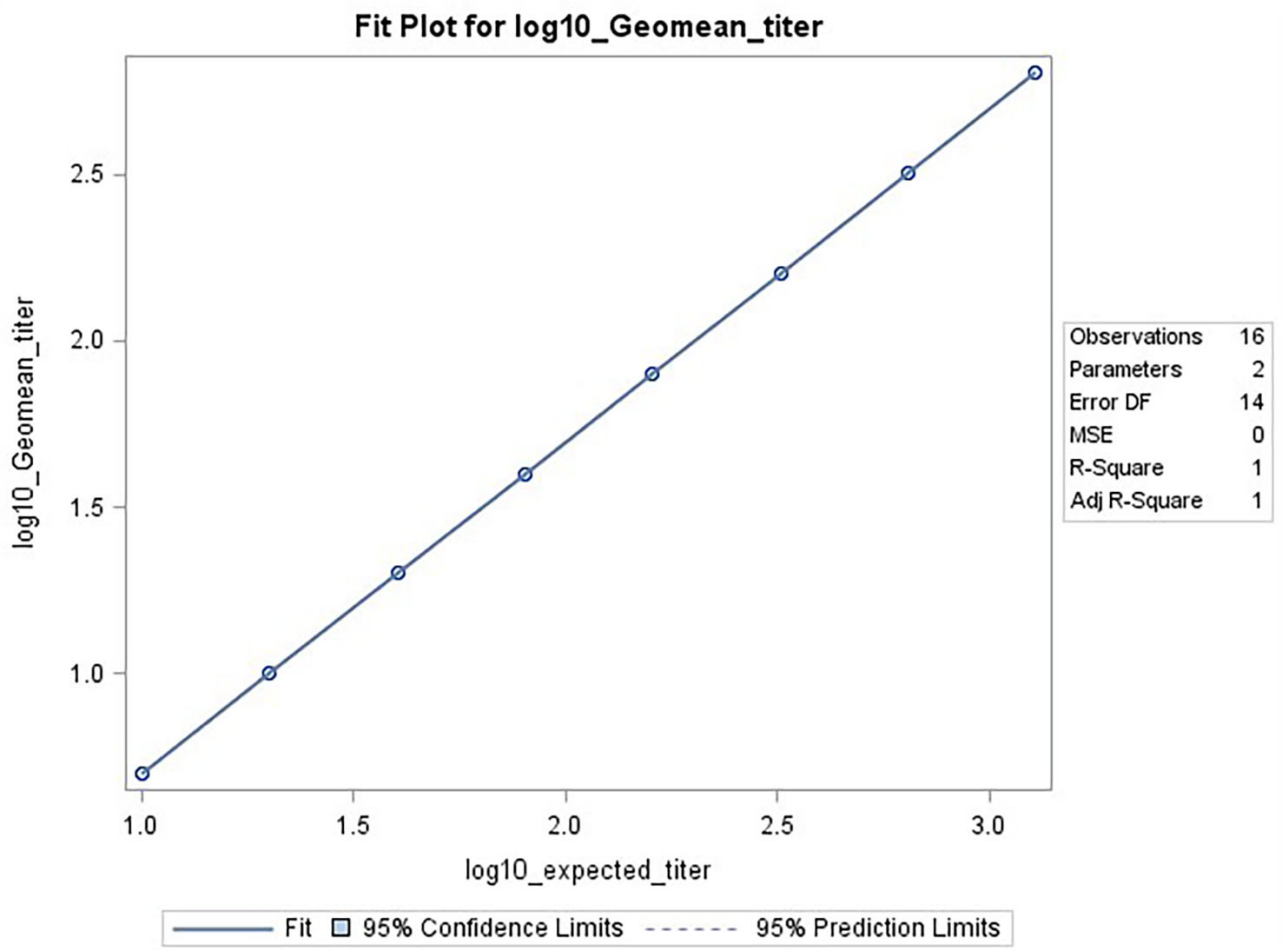
HAI assay qualification on H3N2 influenza strain A/Texas/71/2017, range testing results. The plot shows SAS output from REG procedure, showing the linear regression analysis between the expected [plotted on x-axis] and observed log10 GMTs [plotted on y-axis]. The resulting R^2^ and R was 1.

The data used for the linear regression analysis for linearity testing, which included comparing the observed log10 transformed GMT values with dilution, showed perfect linearity, with a slope of -1 and R^2^ of 1 (**Figures 5A, B**), and acceptable percent linearity (**Table 5**). The linearity of each dilution tested was confirmed by linear regression analysis again, comparing the expected and observed GMTs on log10 transformed data. All dilutions met the linearity acceptance criteria with an R^2^ ≥0.9 (**Figure 6**). Thus, no dilutions were removed from the upper or lower end of the curve for range analysis. The intermediate precision %GCV and %RE values were then calculated on these linearity testing data. The GMTs were equivalent for all replicates tested, resulting in a %GCV of 0% for intermediate precision (**Table 6A**). The data passed the precision acceptance criteria at all 8 dilutions. The expected and observed log10 GMTs differed two-fold at each dilution, resulting in an absolute relative error of 50% at all dilutions (**Table 6B**). This was above the expected %RE for high, medium, and low response levels of ≤ 20% and ≤ 25% for the very low/near-LLOQ value, but within the allowable two-fold assay variation.

**Table 6A:** HAI assay qualification on H3N2 influenza strain A/Texas/71/2017, range testing, %GCV IP results from linearity assays.

Obs	Dilution	Lin_Assay1	Lin_Assay2	%GCV
1	1:10	640	640	0
2	1:20	320	320	0
3	1:40	160	160	0
4	1:80	80	80	0
5	1:160	40	40	0
6	1:320	20	20	0
7	1:640	10	10	0
8	1:1280	5	5	0

Linearity testing data was used for range determination. Table shows the data generated from linearity testing, the GMTs from two assays, performed by two scientists, and the resultant %GCV intermediate precision, by dilution

**Table 6B:** HAI assay qualification on H3N2 influenza strain A/Texas/71/2017, range testing, accuracy %RE results from linearity assays.

Obs	Dilution	log 10 Expected GMT	log10 Observed GMT	%RE
1	1:10	3.10721	2.80618	50
2	1:20	2.80618	2.50515	50
3	1:40	2.50515	2.20412	50
4	1:80	2.20412	1.90309	50
5	1:160	1.90309	1.60206	50
6	1:320	1.60206	1.30103	50
7	1:640	1.30103	1	50
8	1:1280	1	0.69897	50

Table shows the data generated from linearity testing, the log 10 expected GMTs, the log 10 of the mean of the observed GMTs from two assays, performed by two scientists, by dilution, and the absolute value of % RE

Based on the analysis from the linearity assays, all eight dilutions in the dilution series met acceptable linearity, precision, and accuracy criteria. No dilutions needed to be excluded from the dataset to determine the range. The highest and lowest dilutions meeting acceptable linearity, precision and accuracy were used to define the titer calculation range, comprising values between the upper LOQ (ULOQ) and lower LOQ (LLOQ). The expected titer at the highest dilution that was tested (1:1280) was 10. The LLOD was pre-defined as<10, and the LLOQ was set at 10. Usually there was no ULOD set for this assay, as samples with expected high titers can be diluted prior to testing to ensure they fall within the range of the assay. The ULOQ has been set at 1280, the highest titer obtained with the samples available during assay qualification. The resultant range was 10 to 1280 based on the linearity dataset available here. The assay is capable of determining titers up to 2560 and the range of the assay could increase with the availability of more concentrated test samples. Sample with titers greater than the detectable range of the assay are diluted until a titer within the assay dynamic range is obtained.

### Robustness

Robustness is a measure of the capacity of the assay to remain unaffected by small but deliberate variations. The dataset had data from 11 plates across two assays, of which two were tested under ideal experimental conditions. Data from each plate were compared to the reference plates. For two comparisons, at dilution 1:20, and for one comparison at dilution 1:640, there were differences observed in the GMTs between the 2 conditions being tested, changes reagent in lots. At dilution 1:20, in the data generated by one of the two scientists, the plate testing a new lot of RBCs and the plate testing a new lot of A/Texas/71/2017), had a GMT of 905. Reference expected titer for this dilution was 640. At dilution 1:640, GMT was 40 on plate 6 for one of the two scientists. Reference expected titer for this dilution was 20. But these differences were within the acceptable two-fold variation in the assay (**Table 7**). For the remaining three comparisons, there were no differences in GMTs between the reference plate and the plates being compared. The median and mean differences were zero.

**Table 7:** HAI assay qualification on H3N2 influenza strain A/Texas/71/2017, robustness testing, %GCV and %RE results.

Obs	Dilution	Ro	Ro	Ro	Ro	Ro	Ro	Ro	Ro	Ro	Ro	Ro	% GCV	% RE
		Assay1	Assay1	Assay1	Assay1	Assay1	Assay1	Assay2	Assay2	Assay2	Assay2	Assay2		
		P1	P2	P3	P4	P5	P6	P1	P2	P3	P5	P6		
1	1:20	640	640	640	640	905	905	640	640	640	640	640	14	8
2	1:80	160	160	160	160	160	160	160	160	160	160	160	0	0
3	1:320	40	40	40	40	40	40	40	40	40	40	40	0	0
4	1:640	20	20	20	20	20	40	20	20	20	20	20	21	8

The GMTs from robustness testing, by dilution, assay and plate are shown in this table. Total of eleven plates, comprising of two assays performed by two scientists. The %GCV from mixed model analysis and %RE, on log10 GMTs is shown in the last and second last columns of the table

Data from all 11 plates were used for determining precision, on robustness testing. Mixed model analysis was used to assess precision on log10 GMTs. The sum of within and between plate variance was used to calculate the %GCV. To determine accuracy, the mean of data from the two plates run under ideal conditions was used as the expected value. The mean of the data from the remaining nine plates was used as average observed values to calculate % relative error. The range for %GCV was 0 to 21% (**Table 7**). And the range for %RE was 0 to 8% across the four dilutions that were tested (**Table 7**). Precision and accuracy were within acceptable limits for data obtained from the robustness experiments. Based on the above analysis, robustness was met for all conditions that were tested.

### Specificity

The specificity of an assay is the ability to assess unequivocally the analyte in the presence of extraneous components, which may be present. The analytical specificity experiments estimate the systematic error caused by non-analyte materials that may be present in the specimens analyzed. This specificity experiment was performed on two separate occasions using first a 1:20 starting dilution and then a 1:10 starting dilution of serum samples. Geometric mean titers were determined for each specimen. Each assay plate was tested in duplicate by two scientists for a total of four replicates. Geometric mean titers of<20 or<10 dilution were converted to half of the lowest dilution tested in the assay, in this case 10 or 5, respectively, to enable statistical analysis. The response calls (negative and positive) for all (100%) of the samples (**Table 8**).

**Table 8:** HAI assay qualification on H3N2 influenza strain A/Texas/71/2017, specificity testing, and response calls.

Obs	Dilution	Sample Description	Response	Spe_Assay1	Spe_Assay2
1	1:10	7B2 IgG1	GMT	5	5
2	1:10	7B2 IgG1	Response call	Negative	Negative
3	1:10	Ab2210 IgG1	GMT	320	320
4	1:10	Ab2210 IgG1	Response call	Positive	Positive
5	1:10	CDC Serum	GMT	160	160
6	1:10	CDC Serum	Response call	Positive	Positive
7	1:10	FR-1250 Ferret H7N9 Serum	GMT	5	5
8	1:10	FR-1250 Ferret H7N9 Serum	Response call	Negative	Negative
9	1:10	FR-1351 2014 ref Goat (H3) Serum	GMT	160	160
10	1:10	FR-1351 2014 ref Goat (H3) Serum	Response call	Positive	Positive
11	1:10	FR-1377 Normal Goat Serum	GMT	20	10
12	1:10	FR-1377 Normal Goat Serum	Response call	Negative	Negative
13	1:10	FR-1683 2019 ref Goat (H3) Serum	GMT	905	640
14	1:10	FR-1683 2019 ref Goat (H3) Serum	Response call	Positive	Positive
15	1:10	Normal Human Serum	GMT	20	20
16	1:10	Normal Human Serum	Response call	Negative	Negative
17	1:20	7B2 IgG1	GMT	10	10
18	1:20	7B2 IgG1	Response call	Negative	Negative
19	1:20	Ab2210 IgG1	GMT	320	640
20	1:20	Ab2210 IgG1	Response call	Positive	Positive
21	1:20	CDC Serum	GMT	320	160
22	1:20	CDC Serum	Response call	Positive	Positive
23	1:20	FR-1250 Ferret H7N9 Serum	GMT	10	10
24	1:20	FR-1250 Ferret H7N9 Serum	Response call	Negative	Negative
25	1:20	FR-1351 2014 ref Goat (H3) Serum	GMT	160	160
26	1:20	FR-1351 2014 ref Goat (H3) Serum	Response call	Positive	Positive
27	1:20	FR-1377 Normal Goat Serum	GMT	10	10
28	1:20	FR-1377 Normal Goat Serum	Response call	Negative	Negative
29	1:20	FR-1683 2019 ref Goat (H3) Serum	GMT	640	640
30	1:20	FR-1683 2019 ref Goat (H3) Serum	Response call	Positive	Positive
31	1:20	Normal Human Serum	GMT	20	20
32	1:20	Normal Human Serum	Response call	Negative	Negative

GMT and response call (negative or positive), for controls and samples tested for specificity at 1:10 and 1:20 dilutions, are shown in this table, by dilution, sample description, and assay identifier. A GMT of ≥1:40 was designated a positive response call, and a GMT of< 40 designated a negative response call. At both dilutions, all samples met the expected response calls

### Sensitivity and seroprevalence

Eleven out of thirty samples ([(11/30) ×100] 37%), had GMTs below the LOD of 10, 18 of the remaining samples had GMTs of 40 or below. The HAI titers of these samples against A/Texas/71/2017 were not known prior to inclusion in the qualification experiments and as such acceptance criteria was not set. The assay was determined to be sensitive as titers at or near the LOD were detectable. This sample set also confirmed a preliminary indication of a low level of seroprevalence of HAI titers in the population at the time of this study.

### Results from extended qualification for H3N2 influenza strain A/Singapore/INFIMH-16-0019/2016

Extended qualification on the new strain met all pre-specified acceptance criteria (**Supplementary Figure 1**).

#### System suitability criteria

The observed GMTs for positive control Ab2210 IgG1, back titration, IHRS 1:10 and negative control NR-31082 (WNV-E) were within expected limits (**Supplementary Table 1**). The red blood cells controls were all fully precipitated. No plates failed due to assay results outside of the limits of the system suitability criteria.

#### Precision

The %GCVs for intermediate precision and %GCVs for repeatability were 0% for dilutions 1:40 through 1:1280 as all titers were the same between all replicates across all plates. The %GCVs for intermediate precision were 25% and 10% for dilutions 1:10 and 1:20 respectively. The %GCVs for repeatability were 11% and 10% for dilutions 1:10 and 1:20 respectively (**Table 8**). Data was excluded from two plates due to an experimental error for a total of 22 replicates. All dilutions met acceptable criteria for %GCV for intra-operator repeatability and intermediate precision (**Supplementary Tables 2A–C**).

#### Accuracy

The relative error was 0% for dilutions 1:40 through 1:1280. At dilutions 1:10 and 1:20, the relative error was 17% and -3%, respectively. All dilutions met acceptable criteria for accuracy, %RE ≤ 30% (**Supplementary Tables 2A–C**).

#### Linearity

Linear regression analysis was performed between log10 dilution and log10 geometric mean titer. The resulting R and R^2^ was 0.99 (**Supplementary Figure 2**). The percent linearity was 50% to 141% for all combinations that were tested (**Supplementary Table 3**). Percent linearity showed acceptable % linearity values between 50-200%. Linearity was also evaluated by visually assessing the titer versus antibody dilution in the CypherOne instrument graphics (**Supplementary Figure 3**). The slope was -1.01, with a 95% confidence interval (-1.02974, -1.00273), and a p-value<0.0001. Linearity passed with an acceptable R^2^ value ≥0.9.

#### Range, LOD, LOQ

The linearity of each dilution tested was confirmed with linear regression analysis comparing the observed GMT with the expected GMT on log10 transformed data. The resulting R and R^2^ was 0.99, the slope was 1.01 with a 95% confidence interval (1.00273, 1.02974) and a p-value<0.0001 (**Supplementary Figure 4**). All dilutions met the acceptance criteria with an R^2^ ≥0.9, no dilutions were removed from the upper or lower end of the curve as shown in **Figure 6**. Based on the analysis for linearity, precision, and accuracy, all 8 dilutions met acceptable linearity, precision, and accuracy criteria. No dilutions required exclusion from this dataset to determine range. The highest and lowest dilutions meeting acceptable linearity, precision and accuracy were used to define the range comprised of values between upper LOQ (ULOQ) and the lower LOQ (LLOQ). The expected titer at the highest dilution that was tested, 1:1280, was<10. The LLOD was set at<10 and the LLOQ was set at 10, and ULOQ at 640. In general, there was no ULOD set for this assay as samples with expected high titers can be diluted prior to testing to ensure they fall within the range of the assay.

#### Specificity

The assay correctly identified antisera to four homologous strains as positive and antisera to five heterologous strains and one naive serum as negative (**Supplementary Table 4**).

## Discussion

Here, we report on qualification of an HAI assay to quantify strain-specific antibody titers. The matrix effect, precision, accuracy, linearity, range, LOD, LOQ, robustness, specificity, sensitivity and seroprevalence of the assay are successfully demonstrated to ensure reproducible and reliable results (**Figure 2**). Based on these results the HAI assay can be used to test both plasma and serum samples. We have expanded upon previous standardization and qualification efforts (**Table 1**) and have specifically focused on H3N2 influenza strains A/Texas/71/2017 and A/Singapore/INFIMH-16-0019/2016, which are the emphasis of ongoing CIVICs program clinical studies. Further, we employed used guinea pig RBCs as recent H3N2 influenza strains are unable to agglutinate the more commonly used avian RBCs. We implemented the use of CypherOne (19) for objective documentation of agglutination outcome. The use of this equipment removes the need to tilt the assay plate for reading, as well as the subjectivity involved with manually interpreting results by human eye. It also allows for traceability and more rigorous data acquisition.

The methodologies presented here for HAI assay qualification and the ability to extend assay qualification activities to cover additional influenza strains, provides a framework for other researchers to follow and ensure their HAI assays have adequate performance (are precise, accurate and specific) and can be applied reliably for the evaluation of clinical samples. Establishing and employing the use of system suitability criteria, as reported here, for each new strain of virus in evaluation, creates an objective metric that can be used to determine if the assay remains fit for purpose.

An added strength of this work is the use of robust statistical analysis methods and the software used for analysis, compared to previously published literature on HAI qualification. For the statistical analysis on assay linearity, whereas correlations have previously been used (7, 9), here we analyzed linearity by evaluating the correlation co-efficient of the linear regression analysis, the slope, and the 90% confidence interval in 2 ways: linear regression of log 10 observed with log 10 expected values, and a confirmatory analysis of log 10 observed values with the log 10 dilution series. An additional measurement, the percent linearity, which allows for 2-fold change between the subsequent titers, was also utilized. Morokutti et al. (10) performed linear regression and reported R along with slope, and we expand upon this here to include R, R^2^, slope, its 95% confidence interval and p-value of the model. We suggest calculating precision and accuracy with the help of mixed model analysis, which help calculate the %CV as sum of residuals. This calculation provides the geometric coefficient of variation rather than the regular standard deviation divided by mean, the later can underestimate the variance if the covariance structures are not accounted for. The calculation of %GCV (geometric coefficient of variation) instead of a coefficient of variation (%CV), when data are log transformed; or reporting of %GCV (21, 22), alongside reporting number (or percent) of samples within two-fold of GMT for precision testing, is more informative. Previously published manuscripts on qualification of this assay platform have either not stated the software used for data processing and statistical analysis or have used less rigorous software packages for analysis (7, 9–11, 22). Here we have used SAS Analytics Software. This software is most widely used in pharmaceutical development and manufacturing, healthcare, and clinical research companies for analysis of both clinical and non-clinical datasets. It is extensively used for meeting data standards, submissions and reports for the FDA, and within the FDA (23). Resources are available in SAS communities to validate SAS programs as required to meet FDA expectations for trials proceeding to later stages of clinical testing (24).

Our study has several limitations. In this study our primary focus was the qualification of an HAI assay, using guinea pig RBCs and the NA inhibitor oseltamivir. Waldock et al. have shown, based on limited testing, that use of oseltamivir (21) introduce interlaboratory variations. However, harmonization of protocols and the use of reagents from common sources (for example, guinea pig RBCs as used here) can overcome this issue. Assessment of other NA inhibitors, or performing assays with and without oseltamivir to determine its impact on HAI outcome was outside of the scope of this study and could be explored in the future, to determine the impact of this reagent on the parameters described here. An additional limitation to this study is lack of ideal and sufficient quantities of samples with known positive serostatus, as determined in our seroprevalence survey, as well as antibody titers that span the entire range of the assay for testing. As a result, a smaller number of samples was used for the specificity, sensitivity and seroprevalence analysis. This precluded setting a preset acceptance criteria for the latter two, since we did not know the expected serostatus, which would have allowed the comparison of observed responses to expected response status. This limitation was mitigated with use of animal antisera to heterologous and homologous strains to supplement the limited number of human samples available for specificity testing. Despite the limited sample set, the assay was able to differentiate positive and negative samples with 100% accuracy lending confidence to the generated results. A larger sample set was available for testing in the extended qualification which aided in addressing this challenge and again confirmed the specificity of the assay.

In summary, we qualified an HAI assay with the H3N2 A/Texas/71/2017 strain in order to evaluate function and specificity as attributes of potentially protective antibodies in clinical trials. The HAI assay design and statistical methods for data analysis reported here can be utilized for development of additional strain-specific, reproducible HAI assay designs and statistical methods. The use of mixed models analysis to measure random effects when determining precision is recommended to better account for variability in the data. The use of linear regression methods reporting relevant model parameters to depict linearity analysis in details can enable more appropriate and consistent data analysis and comparisons. Improved accuracy and precision of titers with qualified assays incorporating assay automation, suitable statistical analysis methods, reliable analysis software and data analysis pipelines will aid in the use of these output measures as covariates in statistical analysis, such as prediction models and data traceability and will enable comparison across clinical studies. Harmonization studies to confirm the reproducibility of this qualified assay design across labs are in progress and will be of critical importance for the monitoring of assay performance and comparison of HAI titers across laboratories. The method qualification reported here will enable for quantitative head-head comparisons of H3N2 antibody responses across human clinical trials.

## Data availability statement

The raw data supporting the conclusions of this article will be made available by the authors, without undue reservation.

## Author contributions

SS contributed to the design of the work, analyzed, and interpreted data, and helped with writing the manuscript. SG contributed to the conception and design of the work, performed experiments, analyzed, and interpreted data, and helped with writing the manuscript. RO contributed to the design of the work and performed experiments. AS contributed to data processing and helped with writing the manuscript. SM contributed to the conception/design of the work, provided program management support, and helped with writing the manuscript. TO and GS contributed to the conception/design of the work and produced the influenza virus stocks utilized for this study. MS-K, EW, HX, MP, and MM contributed to the conception and design of the work. GT contributed to the conception and design of the work and data interpretation and writing the manuscript. All authors contributed to the article and approved the submitted version.
